# Mobile Phone–based Syndromic Surveillance System, Papua New Guinea

**DOI:** 10.3201/eid1911.121843

**Published:** 2013-11

**Authors:** Alexander Rosewell, Berry Ropa, Heather Randall, Rosheila Dagina, Samuel Hurim, Sibauk Bieb, Siddhartha Datta, Sundar Ramamurthy, Glen Mola, Anthony B. Zwi, Pradeep Ray, C. Raina MacIntyre

**Affiliations:** World Health Organization, Port Moresby, Papua New Guinea (A. Rosewell, H. Randall, S. Datta);; University of New South Wales, Sydney, New South Wales, Australia (A. Rosewell, A.B. Zwi, P. Ray, C.R. MacIntyre);; National Department of Health, Port Moresby (B. Ropa, R. Dagina, S. Hurim, S. Bieb);; Datanets, Port Moresby (S. Ramamurthy);; University of Papua New Guinea, Port Moresby (G. Mola)

**Keywords:** syndromic surveillance, mobile phone, m-health, information and communication technology, ICT, early warning, fragile state, evaluation, Papua New Guinea

## Abstract

The health care system in Papua New Guinea is fragile, and surveillance systems infrequently meet international standards. To strengthen outbreak identification, health authorities piloted a mobile phone–based syndromic surveillance system and used established frameworks to evaluate whether the system was meeting objectives. Stakeholder experience was investigated by using standardized questionnaires and focus groups. Nine sites reported data that included 7 outbreaks and 92 cases of acute watery diarrhea. The new system was more timely (2.4 vs. 84 days), complete (70% vs. 40%), and sensitive (95% vs. 26%) than existing systems. The system was simple, stable, useful, and acceptable; however, feedback and subnational involvement were weak. A simple syndromic surveillance system implemented in a fragile state enabled more timely, complete, and sensitive data reporting for disease risk assessment. Feedback and provincial involvement require improvement. Use of mobile phone technology might improve the timeliness and efficiency of public health surveillance.

Papua New Guinea has been described as a fragile state (*1*). Health care systems in such settings are characterized by limited infrastructure, lack of equity, management capacity issues, and inadequate disease information (*1*). In Papua New Guinea, insufficient investment by government, weak management and leadership capacity, and an inadequate number of health care personnel play a crucial role in the suboptimal performance of the health care system (*2*). Despite these limitations, the country is working toward reaching the minimum requirements of disease surveillance for the International Health Regulations (IHR 2005) (*3*).

Health indicators for Papua New Guinea illustrate some of the country’s challenges: 87% of the population lives in rural areas, the number of primary health care facilities has decreased by 40% over 20 years (*2*), and only 3% of roads are paved. The average life expectancy is 53 years, and the maternal mortality rate of 733/100,000 live births is likely underestimated. Communicable diseases remain the primary causes of illness and death in all age groups, and outbreaks are frequently reported. Lack of health system access and preparedness are particular problems in remote, rural settings (*4*,*5*), whereas migration to informal, periurban settlements and weak infrastructure have been identified as risk factors for disease outbreaks in urban areas (*6*). When compared with other countries in the region, Papua New Guinea often sees more severe effects from outbreaks of commonly occurring pathogens, particularly in remote settings (*4*,*7*–*11*). Special populations, such as internally displaced persons, may be particularly vulnerable to disease outbreaks.

The Papua New Guinea National Health Information System (NHIS) monitors trends for public health syndromes (*12*); in recent years, the Hospital Based Active Surveillance (HBAS) system has been the cornerstone of surveillance for suspected cases of measles, poliomyelitis, and neonatal tetanus (*13*). However, the surveillance system for diseases targeted for elimination or eradication is not achieving globally established performance targets (*14*), and systems for the timely monitoring of endemic diseases, such as diarrheal diseases, are also weak (*15*). Syndromic surveillance offers a useful adjunct to diagnosis-based disease surveillance in developing countries (*16*) and has recently been successfully implemented in the Pacific region (*17*). These systems can be used to detect outbreaks early, to follow the magnitude and geographic distribution of outbreaks, to monitor disease trends, and to provide reassurance that an outbreak has not occurred (*1*).

The use of mobile technology to support the achievement of health objectives has the potential to transform service delivery globally (*18*). Electronic reporting of infectious disease surveillance data has been shown to improve both timeliness and completeness of reporting (*19*). Health information systems are potential benefactors of mobile health solutions for accelerating vital event monitoring in the Asia-Pacific region (*20*). In recent years, greater competition within the communications sector has dramatically increased mobile phone network coverage in Papua New Guinea (*21*). After the delayed detection of serious outbreaks with high mortality rates in rural areas (*4*,*5*,*9*), including an ongoing nationwide cholera outbreak for which the timeliness of surveillance was poor, Papua New Guinea health authorities piloted a mobile phone–based syndromic surveillance system (MOPBASSS) for timely outbreak detection. We describe the system, evaluate its attributes, and determine whether it met its objectives.

## Materials and Methods

### System Descriptions

#### Health System

Papua New Guinea’s population is unevenly distributed among 4 regions; almost 40% of the population lives in the highlands region. The country’s 20 provinces operate within a decentralized health system (*22*). National health authorities have overall responsibility for health care policy and standards, providing technical advice, coordination of the health information system, health planning, and data systems (*22*). Primary health care is the responsibility of provincial governments, and provincial hospitals report to the national level (*2*). Health services are provided through a system of community aid posts, health centers and subcenters, and district and provincial hospitals, as well as a national referral hospital.

#### NHIS

Since 1989, national health authorities in Papua New Guinea have monitored the performance of the health system by using the computerized NHIS (*23*). By 2002, this passive system was centrally managed and regarded as providing quality data for health care monitoring and planning, with links across all health system levels (*12*). Data relevant to health management and disease control programs are collected monthly on paper-based health records from each health center and sent through the district health office to the provincial health office, where the data are entered into a database. The system then calculates percentages using census population data as denominators to provide analysis of disease outbreak and trends (*12*). Hard-copy and electronic data are sent to the national level, where they are re-entered and cleaned before being integrated into the national system. While reporting completeness is strong (*24*), data timeliness and accuracy are not (*25*).

#### HBAS System

Since the late 1990s, a hospital-based surveillance system has been in use in Papua New Guinea (*13*) and has monitored suspected cases of measles, neonatal tetanus, and acute flaccid paralysis (AFP). This zero-reporting system, in which designated reporting sites report even if there are 0 cases, is driven by surveillance officers from the provincial health authorities, who visit the provincial hospitals to review registers and discuss recent patient illness manifestations (signs and symptoms) with the treating clinicians. The forms are compiled monthly and become the documentary evidence to determine if surveillance targets are met and whether poliomyelitis can be excluded as the cause of AFP cases (*13*). The sensitivity of this system is suboptimal, and global performance targets are not routinely met (*14*).

#### Events-based Surveillance System

Information about events (e.g., disease outbreaks, clusters of deaths in humans or animals) that are a potential risk to public health is collected, verified, and assessed by using ad hoc reports transmitted through the health system but also by recording rumors and reports identified through informal channels. Documentation of risk assessments began in 2009.

#### MOPBASSS

The MOPBASSS used in Papua New Guinea was tested in 2 health centers in Port Moresby in 2010, then piloted nationwide during epidemiologic weeks 17–26 during 2011. A 2-stage randomization process first selected the participating provincial, then district, outpatient settings (3 provincial hospitals and 7 district health centers) to participate as reporting sites. The pilot intervention included the provision of data collection tools, a 1-day on-site training, sample collection materials, guidelines, and mobile phones. Ethical approval to conduct the pilot was granted by the Medical Research Advisory Council of Papua New Guinea (MRAC 10.23).

**Figure 1:**
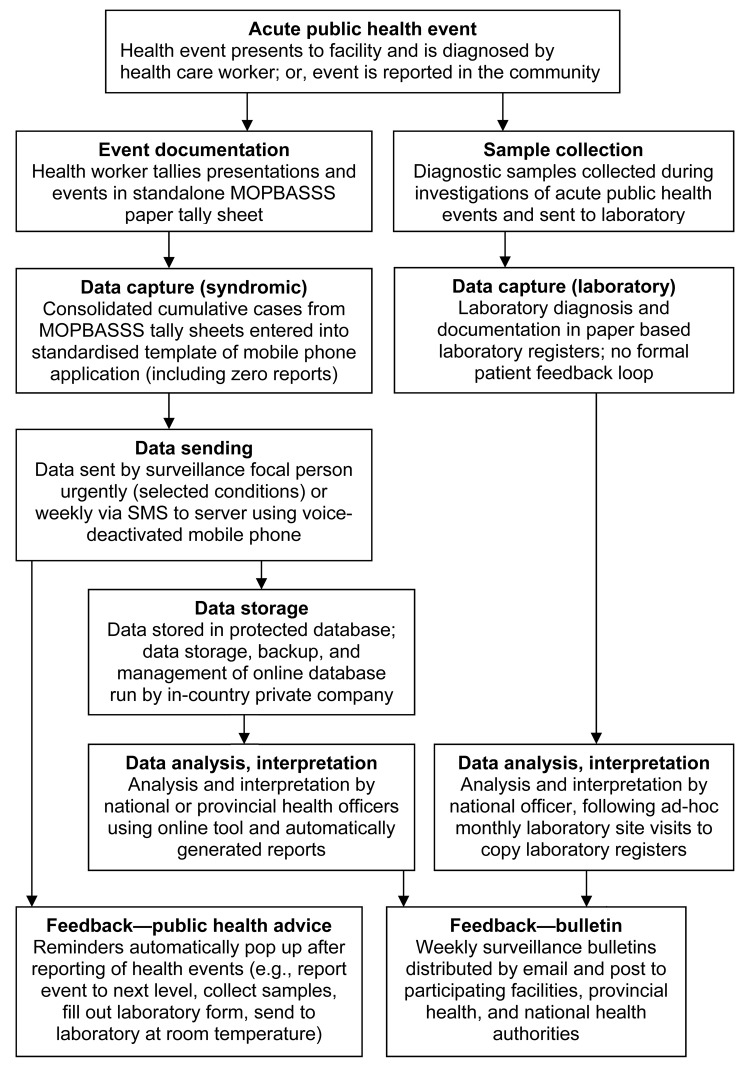
Information flow for mobile phone–based syndromic surveillance system (MOPBASSS) pilot program, Papua New Guinea, 2011. SMS, short message service.

The MOPBASSS information flow is detailed Figure 1. Table 1 lists the system objectives and syndromes under surveillance.

**Table 1:** Objectives and targeted syndromes for mobile phone–based syndromic surveillance system pilot, Papua New Guinea, 2011

System objectives
Identify acute public health events in a timely way
Provide reassurance when events are not identified during an elevated risk period
Establish baseline data for syndromes of public health importance
Strengthen links between clinical services and outbreak response
Complement information generated through the event-based reporting system
Syndromes under surveillance
Influenza-like illness
Acute watery diarrhea
Bloody diarrhea
Prolonged fever
Acute flaccid paralysis
Acute fever and rash
Hemorrhagic fever
Outbreaks or clusters of unexplained severe disease or deaths

### Public Health Event Detection

MOPBASSS data were extracted from the online database, and analyses were performed to describe outbreak detection and user experience. Comparisons were made between MOPBASSS and the NHIS, HBAS, and measles laboratory databases and included the average reporting delay (in days), the completeness of reporting, and the number of measles cases (a frequently reported syndrome common to all 4 systems). The accuracy of data transcription from forms to the database could only be measured at the site that provided usable data at the pilot evaluation meeting. Where no surveillance feedback bulletins were available to make data comparisons with existing systems at week 26, comparisons were made by using data from the next available bulletin so that data could be compared across all systems. Qualitative and quantitative methods were used to evaluate the system, using established frameworks (*26*,*27*).

Nine facilities submitted weekly data through MOPBASSS during the pilot phase. Clinical staff from the 10 sites and public health staff from the 3 provinces participated in focus group discussions. Ten stakeholders (77%) completed the self-administered evaluation survey. Attributes associated with public health event detection were defined as follows:

- Sensitivity—the number of measles cases reported through MOPBASSS compared with the HBAS and NHIS.
- Timeliness—the average number of days reporting delay through MOPBASSS compared with the NHIS.
- Validity—the accuracy of the system to detect outbreaks, measured by comparing reports across systems, including laboratory surveillance data.
- Data quality—the completeness of information recorded in the online database as reported by stakeholders compared with data in the paper-based collection tool.
- Representativeness—the extent to which the system accurately described the distribution of acute public health events in the population.

### System Experience

Qualitative investigations were conducted to describe the system and stakeholder experience by using standardized, self-administered questionnaires and stakeholder focus group discussions conducted by persons experienced in the methodology. Stakeholders included surveillance focal points from the 10 sites (outpatient nurse coordinators and 1 pediatrician), disease control staff from the 3 provincial health offices, and national surveillance staff. Data collection included information on training, the online database, case investigation and diagnosis, reporting using mobile phones, and surveillance guidelines. Attributes associated with system experience were defined as follows:

- Acceptability—the self-reported willingness of stakeholders to further engage with MOPBASSS, as well as indirect measures, including the timeliness and completeness of reporting.
- Stability—the consistency of the system in providing access to public health intelligence, measured by the number of times the system was unable to provide access to data.
- Usefulness—the extent to which stakeholders reported MOPBASSS contributes to public health.
- Portability—user perceptions on how easily the system could be established in another setting.
- Costs—the US dollar amount to establish the piloted system.

## Results

### Public Health Event Detection

#### Sensitivity

Using NHIS as reference, we found that MOPBASSS was more sensitive at detecting measles cases than the HBAS (95% vs. 26%) (Table 2). However, the low number of notifications for the condition “prolonged fever” in MOPBASSS compared with a similar syndrome (malaria) reported in the NHIS indicate the sensitivity for detection of this syndrome may be low.

**Table 2:** Notifications for suspected measles cases during MOPBASSS pilot at 3 provincial hospitals, Papua New Guinea, May–September 2011*

Hospital	Suspected measles notifications			
	MOPBASSS (clinician based)	HBAS (health office based)	NHIS (clinician based)	Fully investigated
A	6 (100)	0	6	0
B	11 (85)	4 (31)	13	0
C	1	1	0	0
Total	18 (95)	5 (26)	19	0

*Values are no. (%). NHIS was used as reference. MOPBASSS, mobile phone–based syndromic surveillance system; HBAS, Hospital Based Active Surveillance system; NHIS, National Health Information System.

#### Timeliness

**Figure 2:**
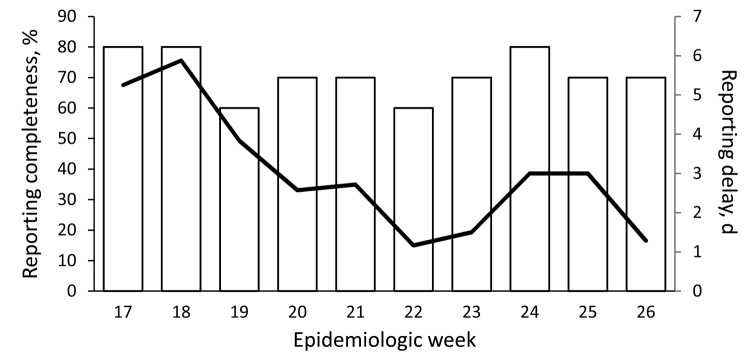
Timeliness (black line) and completeness (white bars) of reporting for mobile phone–based syndromic surveillance system pilot program, Papua New Guinea, 2011.

The MOPBASSS average weekly reporting delay was 2.4 (range 0–52) days (Figure 2), compared with 84 days for the NHIS. Of the 156 MOPBASSS weekly reports, 105 (67%) were submitted on the expected Monday; of these, 57 reports (37%) were submitted by the expected time of 11:00 am . Seven sites (87%) received weekly feedback at least once in the 10-week pilot period; 1 site never received feedback.

#### Validity

The limited microbiological investigation of acute public health events made it difficult to assess the absolute validity of the system. However, the laboratory confirmation of dengue fever virus infection in patients that met the case definition for suspected dengue hemorrhagic fever (DHF) indicates the syndromic data for this condition in this time and place was valid.

#### Data Quality

Although data transcription from the paper-based data collection forms into the phone reporting template and transfer to the database was high quality (98% accuracy), data quality associated with the use of clinical case definitions was not as accurate. The proportion of weekly reports where 0 cases were notified for all syndromes decreased during the pilot, starting at 50% in the first weeks and declining to 15% by the last week of the pilot, which may indicate that clinicians became better at identifying or reporting syndromes. Few data-sending errors occurred, and verification processes ensured no outbreak investigations were instigated erroneously.

#### Representativeness

The 2-stage randomization process to select participating sites and the number of participating sites provides some indication that the timeliness and completeness of reporting that was achieved through MOPBASSS may be generalizable to other provinces. Given that nongovernment facilities in Papua New Guinea are frequently managed more effectively than their government-run counterparts, it is conceivable that this system could function equally well in nongovernment health facilities.

### System Experience

#### Acceptability

All stakeholders reported an interest to continue participation; all but 1 stakeholder reported MOPBASSS was working effectively to detect acute public health events. Stakeholders reported the system was fast, simple, effective, and reliable and enabled the timely initiation of verification, assessment, and response processes. Participation in MOPBASSS was not associated with an excessive time burden, and the program complemented existing systems. Data management was considered simple; it is contracted to the private sector, which removes many person-dependent steps for health authorities. Timely access to data through the Internet-based database was beneficial for national staff, but data access was challenging for provincial staff. The high completeness of reporting through MOPBASSS (70% vs. 40% for HBAS) (Figure 2) and the timeliness and sensitivity of the new system may also reflect its acceptability. The relative validity for outbreak reporting was high; all outbreaks that were identified through alternative systems were also identified through MOPBASSS. However, on several occasions, landline telephone or high-frequency radio might have been the preferred option for providing the initial report for selected conditions.

#### Stability

The system was highly stable during the pilot period, with no reported issues with the online database. The subscriber identification module card from 1 of the 10 mobile phones was misplaced, and reporting ceased at this site for 6 months (week 13 to week 39) until it was reported, the card replaced, and the phone returned. This site also had the weakest mobile phone network coverage.

#### Usefulness

MOPBASSS data was largely used by national health authorities to support inferences about disease patterns that would not have been possible without it; however, stakeholders reported these data were not widely used at the provincial level. Of the 8 clinical sites surveyed, most found feedback either very useful (62%) or useful (25%). Despite some issues with data accuracy, the system provided a certain degree of reassurance that cholera was not circulating at reporting sites during the nationwide outbreak and was considered a measure of the satisfaction of public health decision-makers within the national health authorities.

During the pilot program, MOPBASSS outputs were increasingly used for risk assessments at the weekly surveillance meeting of the national health authorities. The system also facilitated international data reporting to regional monitoring systems. Two weeks after training was conducted at the first established site, a case of hemorrhagic fever was identified, reported, and investigated, which enabled the laboratory confirmation of 3 cases of DHF, which is rarely reported in Papua New Guinea. Similarly, a week after training at a district site, 3 persons meeting the case definition for AFP were identified and reported (not through the mobile phone system); <20 cases of AFP are reported annually from the 20 provinces. Of the 18 cases of suspected measles reported from provincial hospital sites, none were fully investigated.

#### Portability

Stakeholders perceived that the simplicity of the reporting system would likely contribute to its portability and that the system could easily be established among vulnerable populations, such as internally displaced persons or refugees. The system could enable national health authorities to support provincial authorities to work with partners to rapidly establish postdisaster surveillance, particularly if data collection tools were integrated with those of the NHIS.

#### Costs

The pilot intervention cost approximately US$45,000, excluding staff costs. Half the cost was for software development, including the phone template and secure online database. The remaining costs were for investigation materials, mobile phones, and field missions to establish the system. There was no cost to the data provider and no requirement for phone credit.

## Discussion

Creativity and flexibility are crucial when implementing programs that overcome the obstacles and constraints within fragile states (*1*). Establishing MOPBASSS during the nationwide cholera outbreak in Papua New Guinea may have enhanced the program’s acceptability because the threat level and the perceived value of early detection were high, but the program’s attributes likely contributed. We have shown that MOPBASSS reporting was more timely, complete, and sensitive than reporting through existing systems. MOPBASSS reporting was simple, effective, reliable, and acceptable and enabled the routine, systematic, and ongoing reporting of syndromes of public health importance from the district level.

Before the pilot, DHF fever was rarely reported in Papua New Guinea, despite frequent evidence of dengue transmission (*28*). While an association between training and the identification of DHF is not causal, it is likely that the surveillance training, the inclusion of hemorrhagic fever into a routine data collection, and the increased availability of rapid diagnostic tools contributed to the early identification and timely implementation of community level control measures during this outbreak. However, because DHF was not reported through the MOPBASSS, these findings highlight the importance of surveillance training more broadly rather than an advantage of MOPBASSS. In addition, the pre-positioning of dengue rapid diagnostic kits enabled a timely preliminary diagnosis during the outbreak of dengue fever before confirmatory testing and may have demonstrated the utility of rapid tests as an adjunct to MOPBASSS.

The implementation of new surveillance systems can be associated with early confusion regarding clinical case definitions (*29*). During the MOPBASSS reporting pilot, problems were noted with the use of several case definitions, including “prolonged fever,” which gave a lower than expected yield when compared with malaria notifications through the NHIS. Whereas establishing a functional weekly reporting system was the main priority for the pilot, diagnostic accuracy and appropriate use of case definitions can be strengthened through training (*30*) and should be an area of ongoing focus.

Regional measles elimination surveillance standards stipulate that >80% of measles cases should be fully investigated (*31*); however, Papua New Guinea investigates only ≈5% of suspected measles cases. Despite the ability of the MOPBASSS program to provide more timely data on suspected measles, including instant pop-up messages on phone screens reminding clinicians to investigate when selected conditions such as measles are reported, none of the measles cases identified during the pilot were fully investigated. Laboratory support to MOPBASSS when aberrations are detected requires strengthening and may benefit from greater involvement of subnational laboratory staff and the provision of rapid tests for selected conditions.

Geographic representativeness is particularly critical for outbreak detection systems in settings with dispersed populations and challenges to health system access and referral (*32*). Most (87%) of the Papua New Guinea population lives in rural areas, where health system infrastructure and human resources can be limited in general and specifically for outbreak reporting (*33*). When the pilot commenced, cholera had spread widely across the country, but outbreaks had not been identified at participating sites (*9*). The system provided reassurance to national health authorities that no acute watery diarrhea outbreaks were occurring because low case numbers were reported from participating sites during a multijurisdictional and unpredictable cholera outbreak.

Strong linkage between clinical and public health authorities for outbreak detection is emphasized in the regional strategy for strengthening preparedness for emerging diseases (*34*). Syndromic surveillance systems have demonstrated their capacity to strengthen linkage between clinical services and public health authorities (*29*). Such linkages are traditionally weak in Papua New Guinea (*35*), but participation in this pilot program appeared to bring these stakeholders into closer working relationships. MOPBASSS provided opportunities for outpatient nurse clinicians to demonstrate innovation, coordination, and leadership capabilities in making the system work in each setting. These clinicians may be drivers of stronger collaboration on outbreak identification and response with subnational public health authorities. Improving the access to timely data by subnational staff will further reduce barriers to timely public health response and increase ownership of the system, a crucial step toward greater sustainability.

Our evaluation has several limitations. It was not independent, which could introduce measurement bias, and the short intervention period limited our ability to evaluate the flexibility and sustainability of the system. Establishing baseline data for syndromes of public health importance cannot be achieved in a short pilot but might possible with sustained MOPBASSS implementation to capture seasonal and cyclical trends and more informed interpretation of possible aberrations. Data accuracy was only measured at the site that provided data for transcription auditing, but the high accuracy at this site, combined with the user-reported simplicity of the reporting tools and the lack of transcription errors identified during acute public health event verification processes, indicates that data accuracy problem did not affect system sensitivity. Contracting data management to the private sector is simple but can be expensive in a resource-limited setting, so the lack of cost-benefit analysis is also a limitation of our review.

The utility of health information systems to provide data in disasters is being investigated (*36*). We did not measure the flexibility of the system formally, but we believe little additional time, personnel, or funds would be required to accommodate future modifications, such as what types of data are collected and how many data providers are needed for increased population coverage and detection or tracking of low-frequency events. The potential for strengthening health information systems by using mobile phones is not limited to public health event detection; other programs may benefit from the timely sharing of key program data. Adapting the available technology to remotely load data collection templates would enable greater flexibility and would enable additional disease control programs to develop reporting templates for the same mobile device.

Outbreak identification systems that rely on clinician reporting have previously demonstrated their effectiveness (*37*). Automated reporting may decrease the burden on health care and public health workers and enable more complete reporting of potential cases of public health importance (*16*). Data reporting was successfully achieved in MOPBASSS for 2 main reasons: 1) the responsibility for reporting was given to the outpatient department setting that sees clinical cases and was coordinated by a designated leader, and 2) automated reporting system was simple and easy to use. Providing peripheral-level staff with regular feedback is universally recognized as strengthening surveillance programs, as demonstrated by the positive influence of feedback on reporting completeness in the NHIS (*24*). Further consideration is required to ensure feedback can be improved to strengthen the system more consistently and explore how technology may facilitate this process.
